# Predicting Sensory and Affective Tactile Perception from Physical Parameters Obtained by Using a Biomimetic Multimodal Tactile Sensor

**DOI:** 10.3390/s25010147

**Published:** 2024-12-30

**Authors:** Toshiki Ikejima, Koji Mizukoshi, Yoshimune Nonomura

**Affiliations:** 1 POLA Chemical Industries, Inc., Yokohama 244-0812, Kanagawa, Japan; k-mizukoshi@pola.co.jp; 2 Department of Applied Chemistry, Chemical Engineering, and Biochemical Engineering, Graduate School of Science and Engineering, Yamagata University, Yonezawa 992-8510, Yamagata, Japan; nonoy@yz.yamagata-u.ac.jp

**Keywords:** tactile perception, affective, biomimetic multimodal tactile sensor, physical parameters, regression model

## Abstract

Tactile perception plays a crucial role in the perception of products and consumer preferences. This perception process is structured in hierarchical layers comprising a sensory layer (soft and smooth) and an affective layer (comfort and luxury). In this study, we attempted to predict the evaluation score of sensory and affective tactile perceptions of materials using a biomimetic multimodal tactile sensor that mimics the active touch behavior of humans and measures physical parameters such as force, vibration, and temperature. We conducted sensory and affective descriptor evaluations on 32 materials, including cosmetics, textiles, and leather. Using the physical parameters obtained by the biomimetic multimodal tactile sensor as explanatory variables, we predicted the scores of the sensory and affective descriptors in 10 regression models. The bagging regressor demonstrated the best performance, achieving a coefficient of determination (*R*^2^) of >0.6 for fourteen of nineteen sensory and eight of twelve affective descriptors. The present model exhibited particularly high prediction accuracy for sensory descriptors such as “moist” and “elastic”, and for affective descriptors such as “pleasant” and “like”. These findings suggest a method to support efficient tactile design in product development across various industries by predicting tactile descriptor scores using physical parameters from a biomimetic tactile sensor.

## 1. Introduction

Tactile perceptions from touching materials are essential factors that influence the intention to buy or use a product [1,2,3,4,5,6]. These perceptions can be divided into two hierarchical layers: (1) the sensory layer, which involves the perception of physical parameters such as roughness and hardness, and (2) the affective layer, which reflects personal experiences and preferences, such as comfort and luxury [7,8,9]. Previously, tactile perceptions have been evaluated using vocabulary specifically developed to account for these hierarchical structures, providing subjective evaluation indices for product development [8,10]. Recently, we have developed a new tactile vocabulary that considers the hierarchical structure and suitability of descriptors for evaluating a wide range of materials [11]. This new tactile vocabulary enables evaluation across different material groups, although different vocabularies have been used in various industries. This improvement enables cross-industry comparisons of tactile perception, thereby facilitating the development of innovative tactile perceptions, e.g., luxury cosmetic products that mimic the tactile perception of silk fabrics.

In general, tactile evaluations involve having participants touch a material and evaluate it using specific terminology. These processes can be difficult to conduct in product development because of time and cost implications [12]. However, these complications could be significantly reduced if tactile evaluation scores were able to be predicted from physical parameters measured by devices. Several studies have examined the relationship between tactile perception and physical parameters. Chen et al. [13] evaluated 37 plates with different surface treatments using a 20-point scale for seven tactile descriptors: warm–cold, slippery–sticky, smooth–rough, hard–soft, bumpy–flat, wet–dry, and like–dislike. In addition, to calculate the correlation coefficient between tactile scores and physical parameters, they measured surface roughness, friction coefficients, compliance, and temperature using four instruments. Similar studies have examined textiles and confectionery packaging [14,15]. Kikegawa et al. [16] evaluated 11 tactile descriptors, including moistness, for 12 materials, such as powders, leather, and metal, using a visual analog scale (VAS), and confirmed relationships between tactile scores and multiple physical parameters measured with five instruments. These statistical confirmations of tactile scores and physical parameters have been applied in tactile design.

Recently, predictions of tactile perception based on physical parameters have been made using statistical and information methods. Hashim et al. [17] evaluated 26 automotive leathers using a hierarchical tactile vocabulary on a VAS scale, and measured compliance, vibration, temperature, and friction with four instruments to predict sensory and affective perceptions through multiple regression analysis. That model’s coefficient of determination (*R*^2^) ranged from 0.248 to 0.929, indicating high predictive accuracy for tactile descriptors such as temperature, hardness, and preference, but low accuracy for friction-related descriptors such as micro-roughness and macro-roughness. However, their approach was limited and lacked generalizability to other material groups, as it focused on specific materials, such as leather. Furthermore, the time and cost issues were not adequately addressed, as force, vibration, and temperature had to be measured using different instruments to predict multiple tactile descriptors, such as sensations and emotions.

Human fingers with active touch movements (e.g., pressing and stroking) are valuable tools for perceiving and recognizing physical parameters regardless of material type [18]. In recent years, studies have been conducted to estimate tactile perceptions using finger-shaped devices [19,20,21,22]. One such example is the Toccare system, which uses the BioTac [21], a finger-shaped device that simulates human tactile movements to touch materials [22]. This system allows the measurement of multiple physical parameters, such as force, vibration, and thermal stimulation, with a single device, without being limited by the type of material. Fishel et al. [23] used a finger-shaped device to measure force, vibration, and temperature across 117 different materials, with the results confirming the device’s high accuracy in evaluating roughness and friction. Systems that include this biomimetic multimodal tactile sensor have been used for evaluating the tactile sensations of liquid cosmetics on hair [24] and the surface roughness of plates with different treatments [25]. Furthermore, it has already been established that the BioTac can estimate sensory tactile perceptions [26]. However, even with a finger-shaped device, human affective tactile perceptions have not yet been accurately predicted. Affective tactile perceptions are complex and are composed of multiple physical properties and sensory tactile perceptions [13]. Therefore, we hypothesized that the Toccare system, which uses the multimodal tactile sensor to perform measurements simulating human touch movements, would also be capable of predicting affective tactile perceptions.

Given this background, in the present study, we focused on the sensory and affective tactile perceptions of various materials—primarily soft materials such as cosmetics, textiles, leather, wood, and rubber. Using a system that includes a biomimetic multimodal tactile sensor [22], we sought to obtain physical parameters through integrated measurements that simulate human touch movements. These physical parameters are the explanatory variables to be predicted, whereas the evaluation scores of our newly developed tactile vocabulary [11] are the objective variables. By examining 10 regression models to determine the model with the highest accuracy across various tactile descriptors, we aimed to predict each sensory and affective tactile perception more efficiently and affordably than conventional methods by using physical parameters. Our findings could be expected to be useful for more efficient tactile design and the creation of new tactile sensations in less time and at a lower cost compared with conventional methods.

## 2. Materials and Methods

### 2.1. Materials

In a previous study, 32 materials were evaluated (Table 1) [11]. That study primarily included soft materials that people frequently contact in daily life, such as cosmetics, fabrics, polymers, paper, rubber, wood, metal, tiles, clay, adhesive tape, and leather. All objects were prepared in 10 cm × 10 cm square shapes. Cosmetics, liquid lotions, serums, and creams were evaluated under two conditions. In the first method, the study subjects evaluated tactile perceptions when 0.2 g of a cosmetic product was applied directly onto a 10 cm × 10 cm square piece of artificial leather. In the second method, the evaluation occurred at 5 min after application.

### 2.2. Tactile Vocabulary

In this study, a tactile vocabulary comprising 31 descriptors was organized into two hierarchical layers: sensory and affective (Table 2) [11]. The sensory layer was composed of 19 descriptors describing the physical parameters of materials such as soft, smooth, and warm sensations, while the affective layer was composed of 12 descriptors expressing impressions and emotions evoked by the tactile perception, such as pleasant, like, and luxury.

### 2.3. Participants

The study participants were 64 Japanese native speakers (50% male; age range, 20–59 years) with an equal age distribution. All participants were informed about the content of the experiment and provided written consent to participate in the study and for their data to be used for research purposes. This study was reviewed and approved in advance by the Ethics Committee of POLA Chemical Industries, Inc. (Yokohama, Japan) (protocol code 2020-F-121, 21 October 2020).

### 2.4. Evaluation of Tactile Vocabulary Scores

In this study, the intensity scores for tactile vocabulary obtained in a previous study were used as the tactile perception scores [11]. The scores were defined as the median of the raw evaluation scores of the 64 subjects for each material. The participants touched one of each material and rated each tactile perception on a monitor using a six-point Likert-type scale, from 1, “not felt at all” to 6, “felt very strongly”. These evaluations were conducted under conditions where the participants could visually observe the materials to replicate the tactile evaluation humans experience in daily life. The subjects touched the surface of each material with the index finger of their nondominant hand. The subjects cleaned their fingers with wet wipes and tissues before touching different materials to ensure uniform finger conditions. The order of the material presentation and vocabulary was randomized. Each subject rated 10 materials each so that 20 people rated each material.

### 2.5. Evaluation of Physical Parameters

**Figure 1:**
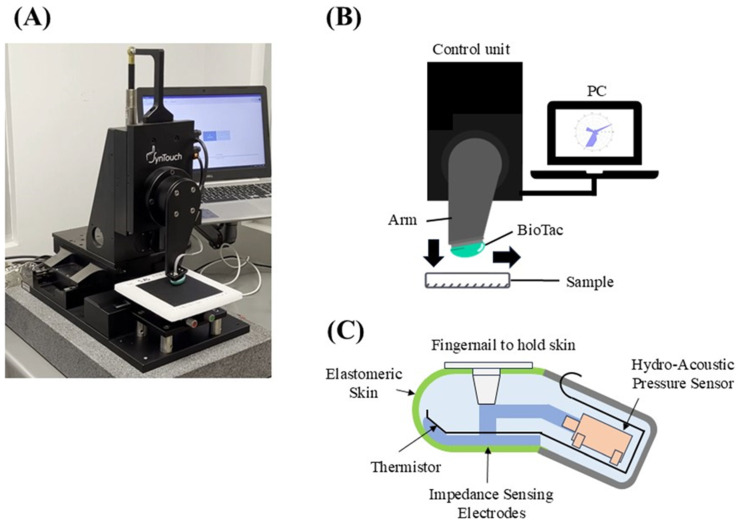
Overview of the Toccare system and biomimetic multimodal tactile sensor. (**A**) Experimental apparatus, (**B**) measuring mechanism, and (**C**) schematic of the BioTac finger-shaped tactile sensor.

The physical parameters of the materials were evaluated using the Toccare system [22], which incorporates the BioTac biomimetic multimodal tactile sensor [21]. Figure 1 shows an overview of the system and device. The device was equipped with internal sensors, including a pressure sensor, electrodes, and a thermistor. The finger-shaped device is covered with an elastomer-based artificial skin filled with incompressible conductive fluid. Force is estimated based on voltage changes caused by the deformation of the artificial skin and the internal fluid when in contact with a material [27]. Micro-vibrations generated by contact with the material propagate through the internal fluid and reach a hydro-acoustic pressure sensor embedded in the sensor. The fingerprint pattern engraved on the surface of the artificial skin amplifies the vibration amplitude, enabling the detection of micro-vibrations [28]. The internal fluid is heated to human body temperature by a heater embedded in the electronic circuit. This ensures that, upon contact with a material, the sensor generates thermal conduction similar to human touch, allowing the estimation of the material’s thermal properties. From these measurement data, the original physical parameters listed in Table 3 were predicted using Bayesian exploration, resulting in 15 items similar to human tactile perception [24]. The 15 physical parameters were categorized as follows: friction (fST, fRS, and aTK), macro-roughness (mTX, mCO, and mRG), micro-roughness (μRO and μCO), thermal properties (tCO and tPR), and compliance (cCM, cDF, cDP, cRX, and cYD). Although the default lower and upper limits for each parameter were 0 and 100, respectively, scores of ≥100 were calculated in some cases [23,29]. Several defined movements—short- or long-distance sliding and pushing of the flat surface with the device—were performed in the evaluation of the physical parameters. The mean was selected as the physical parameter for each material when measurements were conducted five times.

### 2.6. Prediction of Tactile Perception Scores

Regression models for affective or sensory descriptors were examined using subject median scores for 15 physical parameters, 19 sensory tactile descriptors, and 12 affective tactile descriptors of 32 materials. A total of 10 estimation models were used, with eight regression models including AutoGluon [30], CatBoost [31], and Bagging Regressor [32]. The 10 regression models included four linear regression models: Linear Regression, Lasso, Ridge, and Elastic Net. As for nonlinear models, they consisted of SVR, Decision Tree, Random Forest, and ensemble learning models such as Gradient Boosting, CatBoost, and Bagging Regressor. Linear Regression is the most basic regression model, which represents the relationship between explanatory variables and the target variable using a linear function. Lasso improves model sparsity by adding an L1 regularization term to linear regression, which shrinks coefficients close to zero. Ridge prevents overfitting by adding an L2 regularization term to linear regression. Elastic Net combines Lasso and Ridge, incorporating both L1 and L2 regularization techniques. SVR (Support Vector Regression) applies Support Vector Machine (SVM) concepts to regression problems, performing regression by keeping data within a margin. The Decision Tree predicts outcomes by splitting data into multiple conditions, represented in a tree structure. Random Forest is an ensemble learning method that combines multiple decision trees to make predictions. Gradient Boosting constructs decision trees sequentially, learning iteratively to minimize prediction errors. CatBoost is a variant of gradient boosting, specialized for handling categorical data efficiently. The Bagging Regressor uses Bootstrap Aggregating to improve the robustness and accuracy of predictions. It works by creating multiple subsets of the original dataset through random sampling with replacement. Each subset is then used to train an independent model, typically of the same type, such as decision trees. The final prediction is obtained by averaging the predictions of all individual models. This approach reduces variance and prevents overfitting, which are common issues with individual models.

For sensory perception, the 15 physical parameters for the 32 materials were used as explanatory variables to create models for the 19 sensory scores. For affective scores, the same 15 physical parameters were used as explanatory variables to create models for 12 affective tactile scores. During model construction, the dataset was divided into training and test data at a ratio of 8:2. Hyperparameters were tuned to determine the best conditions for each target variable and maximize accuracy. This process was repeated five times with different data splits for cross-validation. Model accuracy was evaluated using *R*^2^. For each target variable, the *R*^2^ value of the model for the test data was evaluated, and the average *R*^2^ value across the five repetitions was used to represent the model accuracy. The criteria for model achievement were based on the accuracy reported in a previous study [17], with an average *R*^2^ value of ≥0.6. Regression models were developed using the Python 3.9 libraries autogluon.tabular, catboost, and sklearn.ensemble. Furthermore, the contribution of explanatory variables was calculated for each sensory and affective tactile perception prediction model.

## 3. Results

### 3.1. Prediction Models for Tactile Perception

**Figure 2:**
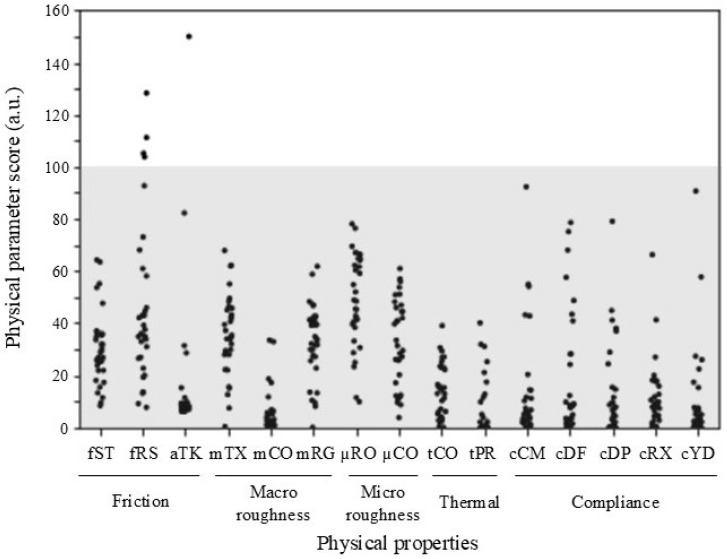
Variation in physical parameters for materials. Gray zone is basic range measurement range. Each dot represents the score of a single material.

The 15 physical parameters for each material are shown in Figure 2 and Table S1, the median of the participant sensory ratings for 19 descriptors in Table S2, and the median participant affective ratings for 12 descriptors in Table S3. Table 4 presents the test results of 10 regression models using the 15 physical parameters to predict each sensory and affective descriptor, consisting of four linear models, such as linear regression and ridge regression, and six nonlinear models, such as Support Vector Regression (SVR) and the Decision Tree. For each model, the number indicates the number of tactile descriptors for which *R*^2^ was ≥0.6 in the 19 sensory and 12 affective descriptors.

Among the linear models, Elastic Net had the highest number of descriptors with *R*^2^ values > 0.6: fourteen of nineteen for sensory descriptors and four of twelve for affective descriptors. By contrast, Ridge Regression had the lowest number, with only four of thirty-one descriptors higher than the threshold. For the nonlinear models, the Bagging Regressor had the highest number of descriptors with *R*^2^ values > 0.6: fourteen of nineteen for sensory descriptors and eight of twelve for affective descriptors. By contrast, SVR had the lowest number: three sensory descriptors and three affective descriptors. Overall, the Bagging Regressor was the top-performing model, covering the highest number of tactile descriptors with *R*^2^ values > 0.6 across all models for both sensory and affective descriptors.

### 3.2. Accuracy of Tactile Perception Prediction

The *R*^2^ and contribution of explanatory variables for the Bagging Regressor test data for the 19 sensory descriptors is shown in Table 5A. For the six descriptors representing moistness, the *R*^2^ values were 0.911 (slimy), 0.904 (soggy), 0.850 (sticky), 0.848 (wet), 0.823 (moist), and 0.748 (dry), indicating generally high estimation accuracy. For the two descriptors representing temperature, the *R*^2^ values were 0.901 (cold) and 0.337 (warm), showing high and low estimation accuracy, respectively. For the four descriptors representing elasticity and softness, the *R*^2^ values were 0.869 (elastic), 0.829 (soft), 0.796 (firm), and 0.767 (hard), showing generally high estimation accuracy. For the seven descriptors representing roughness and friction, the *R*^2^ values were 0.660 (bulky), 0.607 (slippery), 0.603 (rough), 0.592 (smooth), 0.487 (scratch), 0.484 (sleek), and 0.292 (bumpy). While three of the descriptors exceeded an *R*^2^ value of 0.6, four descriptors did not. The contribution of explanatory variables was identified for each tactile prediction model, listing the top three contributing physical properties out of the 15 parameters.

The results for the bagging regressor in predicting the 12 affective tactile descriptors are shown in Table 5B. The *R*^2^ values for the test data of each model were 0.829 (unpleasant), 0.705 (calm), and 0.627 (pleasant) for three descriptors related to comfort, discomfort, and arousal. For the two descriptors representing preference for a material, the *R*^2^ values were 0.676 (like) and 0.489 (dislike). For the seven descriptors representing impressions of the material, the *R*^2^ values were 0.774 (interest), 0.674 (luxury), 0.624 (fine), 0.622 (friendly), 0.516 (comfort), 0.485 (delicate), and 0.304 (slight warmth).

**Figure 3:**
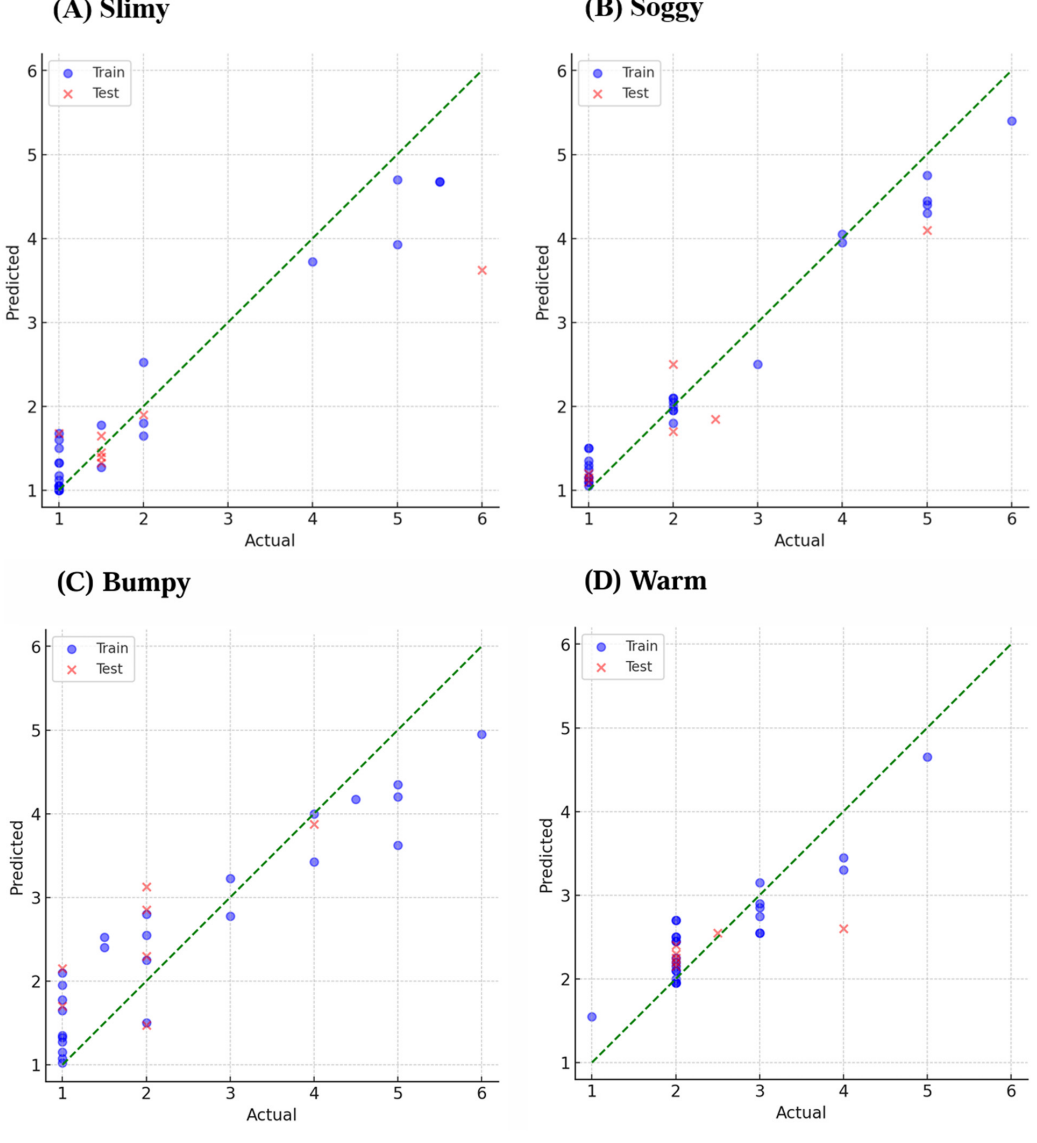
Prediction model for the representative sensory descriptors: (**A**) slimy, (**B**) soggy, (**C**) bumpy, and (**D**) warm. Blue circles indicate training data, and red crosses indicate test data.

**Figure 4:**
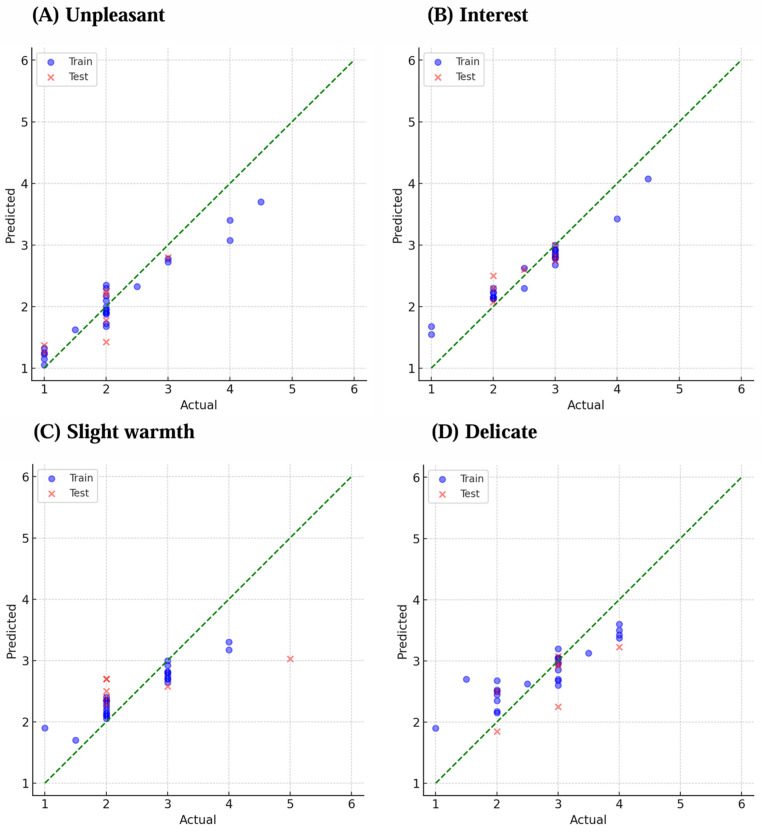
Prediction model for the representative affective descriptors: (**A**) unpleasant, (**B**) interest, (**C**) slight warmth, and (**D**) delicate. Blue circles indicate training data, and red crosses indicate test data.

Scatterplots of the predicted and actual scores for the two tactile descriptors with the highest *R*^2^ values from the sensory and affective layers are shown in Figure 3 and Figure 4, respectively. Circles represent the training data, crosses represent the test data, and the dotted line indicates the one-to-one relationship between predicted and actual scores. Figure 3 shows the representative results for slimy and soggy as higher *R*^2^, and for bumpy and warm, which showed lower *R*^2^ for sensory descriptors. Figure 4 shows the representative results for unpleasant and interest as higher *R*^2^, and for slight warmth and delicate, which showed lower *R*^2^ for affective descriptors. For these tactile descriptors with higher *R*^2^, within the score range of 1–6, the predicted values increase as the actual values increase for both the training and test data, indicating a linear relationship.

## 4. Discussion

In this study, we evaluated 15 physical parameters of a diverse set of 32 materials, including cosmetics, textiles, leather, wood, metal, and rubber, using a system that included a biomimetic multimodal tactile sensor. Based on the obtained parameters, we succeeded in predicting accurate scores for 14 sensory descriptors and eight affective descriptors by the Bagging Regressor. Therefore, the present method appears to be useful for solving cost and time issues, as physical parameters of materials in different fields can be acquired by a single device and a wide range of sensory and affective perceptions can be predicted. The 32 materials included 26 soft materials selected from the 117 previously examined by Fishel et al. [23]. We added liquid materials such as cosmetics to broaden the range of commonly used materials. Previous studies have shown that a system with a biomimetic multimodal tactile sensor is suitable for the evaluation of a wide range of materials [24,25,26,33,34,35].

In this study, we examined 10 regression models using 15 physical parameters as explanatory variables to predict 19 sensory and 12 affective descriptors. We evaluated the average coefficient of determination for each tactile descriptor. We set the threshold for prediction as an average coefficient of determination of ≥0.6 based on the accuracy levels reported in a previous study [17]. Among the models, the largest number of tactile descriptors satisfied the threshold condition in the case of the Bagging Regressor. Previously, linear models were frequently used to predict tactile perceptions based on physical parameters [17]. Sagara et al. [36] recommended nonlinear models for predicting tactile perceptions, as human tactile characteristics are inherently nonlinear, and demonstrated that nonlinear models improve predictive accuracy compared with linear models. The bagging regressor is one of the nonlinear and ensemble learning methods. Ensemble learning includes techniques such as bagging, boosting, and stacking, all of which combine multiple models to achieve high accuracy and generalization [37]. Among these, the Bagging Regressor adopts the bagging approach, where multiple models are built, and their prediction results are averaged to make the final estimation. This model has been widely applied in fields such as real estate price prediction and economic growth forecasting [38,39,40,41,42,43]. The reason why the case using the bagging regressor achieved the highest accuracy in this study is likely because the approach of averaging multiple models was effective in addressing challenges such as the nonlinearity of tactile perception and noise caused by individual differences. By exploring such methods, we can improve the accuracy of tactile prediction and potentially show the mechanisms behind tactile perceptions.

Traditionally, sensory perception is known to be divided into five haptic dimensions [44,45,46,47,48]. As shown in Table 5A, the present model achieved high predictive accuracy for wetness and hardness and cold sensations. In addition, warmth was more difficult and roughness and bumpiness were more challenging to predict. The high predictive accuracy for wetness and hardness can be attributed to the larger variability in tactile evaluation scores among the materials. For cold sensations, the variability in evaluation scores was higher, particularly for liquid cosmetics. However, warmth showed smaller variability across materials, and the threshold between individual materials was insufficient; these trends resulted in lower predictive accuracy. For descriptors related to roughness and bumpiness, previous studies have shown that descriptors of roughness are correlated with the surface roughness parameter (*R_a_*) [16]. Therefore, the physical measurements obtained in the present study may not fully capture the physical information related to the roughness perception. In the future, an increase in the diversity and number of materials could improve the prediction accuracy. Furthermore, the extraction of more relevant features related to roughness and unevenness from the vibration information obtained by the biomimetic multimodal tactile sensor will be important.

As shown in Table 5B, the predictive accuracy for affective descriptors was generally lower than that for sensory descriptors: a coefficient of determination of ≥0.6 was achieved for eight affective descriptors. Traditionally, attempts to estimate sensory tactile perceptions using other finger-shaped sensors or the BioTac have been made [21,22,24,26]. The ability to accurately predict affective tactile perceptions, as achieved in this study, holds significant importance for tactile perception prediction. Sufficient predictive accuracy was obtained for descriptors related to valence and arousal in Russell’s circumplex model of affection (“unpleasant”, “pleasant”, and “calm”) [49]. Furthermore, descriptors associated with personal preference (“like”) and those reflecting impressions of materials (“interest”, “luxury”, “fine”, and “friendly”) also showed high predictive accuracy. Affective tactile perception can be attributed to the complex interplay of multiple factors [13]. Furthermore, affective tactile perception is thought to be based on the hierarchical structure of physical parameters and sensory tactile perception [7]. The BioTac, being a multimodal sensor, simultaneously measures force, vibration, and temperature, accounting for the impact of each parameter. These characteristics of the tactile sensor may make it particularly effective for estimating complex tactile perceptions. However, the predictive accuracy was lower for descriptors such as “comfort”, “dislike”, “delicate”, and “slight warmth”. The low accuracy could be attributed to the uneven distribution of evaluation scores within the materials and the possibility that the physical measurements obtained in the present study may not fully capture the physical information related to above affective perceptions. Indeed, Figure 2 showed that parameters such as mCO, which represents macro-roughness, and tCO and tPR, which relate to temperature, have relatively small variance. As shown in Table 5, tactile prediction models with lower accuracy often included explanatory variables with small variance among their contributing physical parameters. For the affective descriptors that could not be predicted in this study, the features related to macro-roughness or thermal properties may have played an important role. Therefore, further investigation with a larger variety and quantity of materials is necessary to improve the prediction of affective descriptors. Additionally, affective perceptions are influenced by cultural backgrounds [50,51]. Therefore, further studies are needed to predict evaluation scores for participants with different cultural backgrounds or who do not speak Japanese as their native language. Furthermore, the variety and number of materials could be improved. An increase in the variation of materials could enhance the models that have lower predictive accuracy. The individual differences in evaluation scores were greater for affective tactile than for sensory descriptors. Sagara et al. [36] demonstrated that clustering participants based on their preferences and experiences improved predictive accuracy. In the present study, clustering participants has a potential for enabling the development of products tailored more closely to consumer preferences.

## 5. Conclusions

In this study, we examined a prediction model for tactile evaluation score by using a tactile vocabulary that enables tactile evaluation across various materials, guided by a suitability indicator, and by employing a biomimetic multimodal tactile sensor. This system mimicked human touch movements to measure physical parameters related to force, vibration, and temperature. The results confirmed that the model could predict sensory perceptions such as moistness, hardness, elasticity, and coldness, as well as affective perceptions such as pleasantness, unpleasantness, calmness, and preference. Based on our findings, more efficient tactile design and novel tactile experiences could be possible in the development of materials and products in various industrial sectors.

## Figures and Tables

**Table 1:** List of materials used in this experiment.

No.	Materials	No.	Materials
1	Lotion *^1^	17	Broad cloth
2	Serum *^1^	18	Cotton
3	Cream *^1^	19	Nylon
4	Lotion (after application) *^2^	20	Cashmere
5	Serum (after application) *^2^	21	Leather
6	Cream (after application) *^2^	22	Styrene foam
7	Sticky tape	23	Western paper
8	Slime	24	Japanese paper
9	Clay	25	Wood plate
10	Artificial skin	26	Stainless plate
11	Fur	27	Polishing sponge
12	Mesh (rough)	28	Cork
13	Mesh (fine)	29	Convex rubber
14	Sponge rubber	30	Tile
15	Rubber	31	Acrylic plate
16	Low rebound sponge	32	Artificial leather

*^1^: Material was applied to artificial leather (No. 32). *^2^: Material was applied to artificial leather (No. 32) and left for 5 min.

**Table 2:** List of descriptors in sensory and affective tactile vocabulary.

(A) Sensory Descriptors	
No.	Sensory Descriptors
1	Bulky
2	Bumpy
3	Cold
4	Dry
5	Elastic
6	Firm
7	Hard
8	Moist
9	Rough
10	Scratch
11	Sleek
12	Slimy
13	Slippery
14	Smooth
15	Soft
16	Soggy
17	Sticky
18	Warm
19	Wet
**(B) Affective Descriptors**	
**No.**	**Affective Descriptors**
1	Calm
2	Comfort
3	Delicate
4	Dislike
5	Fine
6	Friendly
7	Interest
8	Like
9	Luxury
10	Pleasant
11	Slight warmth
12	Unpleasant

**Table 3:** Fifteen different physical parameters obtained by the Toccare system.

Sensors	Genres	Items	Details
Pressure	Friction	fST	Effort required to initiate sliding on a surface
		fRS	Effort required to continue sliding on a surface
		aTK	Effort required to break contact with a surface
	Macro-roughness	mTX	Intensity of large features (>1 mm spacing)
		mCO	Perceived spacing of large features (>1 mm)
		mRG	Perceived uniformity of large features (>1 mm)
	Micro-roughness	μRO	Intensity of small features (<1 mm spacing)
		μCO	Perceived spacing of small features (<1 mm)
Thermistor	Thermal	tCO	Initial rate that a surface draws heat from the fingertip
		tPR	Extent that a surface continues drawing heat from the fingertip
Electrodes	Compliance	cCM	Degree that a surface deforms under pressure
		cDF	Degree of surface wrap around the fingertip when being deformed
		cDP	Speed that a surface returns to its original shape after being deformed
		cRX	Degree to which a surface stops pushing back after being deformed
		cYD	Degree to which a surface remains deformed after being pressed

**Table 4:** Comparison of prediction models for tactile descriptors.

Model	Feature	Sensory Descriptors	Affective Descriptors	Total Descriptors
Bagging Regressor	Nonlinear (ensemble)	14/19	8/12	22/31
Decision Tree	Nonlinear (decision tree)	14/19	4/12	18/31
Elastic Net	Linear	12/19	5/12	17/31
Random Forest	Nonlinear (decision tree)	11/19	6/12	17/31
Lasso	Linear	10/19	5/12	15/31
Gradient Boosting	Nonlinear (ensemble)	11/19	2/12	13/31
CatBoost	Nonlinear (ensemble)	8/19	3/12	11/31
Linear Regression	Linear	4/19	4/12	8/31
Ridge	Linear	4/19	4/12	8/31
SVR	Nonlinear	3/19	3/12	6/31

The average coefficient of determination (*R*^2^) for each model was calculated for each objective variable. The numbers of sensory and affective descriptors represent how many objective variables had an average *R*^2^ of ≥0.6 in relation to the total number of objective variables. The total descriptors are the sum of the numbers for sensory and affective descriptors that had an average *R*^2^ of ≥0.6 across all descriptors.

**Table 5:** Accuracy of the tactile prediction model (Bagging Regressor).

(A) Sensory Descriptors		
Sensory Descriptors	Coefficient of Determination	Contribution of Explanatory Variables
**Slimy**	**0.911**	**fRS, µCO, cDP**
**Soggy**	**0.904**	**aTK, cDP, tCO**
**Cold**	**0.901**	**tCO, cRX, cYD**
**Elastic**	**0.869**	**cRX, cDP, aTK**
**Sticky**	**0.850**	**aTK, cDP, fRS**
**Wet**	**0.848**	**fRS, cDP, tCO**
**Soft**	**0.829**	**cYD, cDP, cCM**
**Moist**	**0.823**	**cDP, cYD, fST**
**Firm**	**0.796**	**cDP, fST, cDF**
**Hard**	**0.767**	**fRS, tPR, cDF**
**Dry**	**0.748**	**cDP, fRS, µCO**
**Bulky**	**0.660**	**µCO, cDP, µRO**
**Slippery**	**0.607**	**µCO, cCM, mCO**
**Rough**	**0.603**	**cDP, µCO, tCO**
Smooth	0.592	µCO, fST, µRO
Scratch	0.487	µRO, mCO, cRX
Sleek	0.484	cDF, mCO, µRO
Warm	0.337	tCO, mRG, mCO
Bumpy	0.292	mTX, fRS, fST
**(B) Affective Descriptors**		
**Affective** **Descriptors**	**Coefficient of Determination**	**Contribution of Explanatory Variables**
**Unpleasant**	**0.829**	**aTK, µRO, cDF**
**Interest**	**0.774**	**fRS, cDP, aTK**
**Calm**	**0.705**	**µCO, cYD, mRG**
**Like**	**0.676**	**cCM, µRO, fRS**
**Luxury**	**0.674**	**µRO, fRS, cYD**
**Pleasant**	**0.627**	**µRO, aTK, cYD**
**Fine**	**0.624**	**µCO, µRO, aTK**
**Friendly**	**0.622**	**µCO, tCO, µRO**
Comfort	0.516	µCO, tPR, µRO
Dislike	0.489	tCO, aTK, cRX
Delicate	0.485	tCO, aTK, tPR
Slight warmth	0.304	tCO, tPR, mRG

The average *R*^2^ of each tactile descriptor is shown. Bold font indicates an average *R*^2^ of ≥0.6. The contribution of explanatory variables is listed for each tactile prediction model, with the top three variables in order of their contribution.

## Data Availability

The data that support the findings of this study are available on request from the corresponding author.

## Supplementary Materials

The following supporting information can be downloaded at https://www.mdpi.com/article/10.3390/s25010147/s1. Table S1: Physical parameters of each material. Table S2: Sensory descriptor scores for each material. Table S3: Affective descriptor scores for each material.
