# Supplementary material for: Predicting Sensory and Affective Tactile Perception from Physical Parameters Obtained by Using a Biomimetic Multimodal Tactile Sensor
Source: Sensors (Basel). 2024 Dec 30;25(1):147. doi: 10.3390/s25010147 (PMC11723034; doi:10.3390/s25010147)
Supplement: Supplementary file 1 [file sensors-25-00147-s001.zip › Table S1.pdf]

Table S1. Physical parameters of each material.

Physical parameters /materials	fST	fRS	aTK	mTX	mCO	mRG	μRO	μCO	tCO	tPR	cCM	cDF	cDP	cRX	cYD
Cream	9.45	7.68	7.72	37.13	2.08	48.27	28.64	12.39	15.71	0	5.97	7.71	11.51	10.48	7.62
Serum (after application)	18.01	9.1	6.06	21.99	0.43	22.73	30.59	31.31	16.4	2.04	6.69	9.05	8.66	9.78	3.1
Serum	21.87	13.09	6.92	39.44	4.86	39.18	60.34	26.06	26.9	0	5.65	8.38	9.39	11.73	4.94
Cream (after application)	11.39	13.59	15.19	35.38	1.71	46.77	33.13	17.11	11.31	0	5.73	9.72	9.52	7.3	2.9
Lotion (after application)	25.42	19.23	7.41	43.25	6.73	42.65	66.75	25.7	23.07	0	5.08	8.05	7.85	8.15	4.76
Lotion	26.76	19.95	6.51	42.13	5.08	41.42	66.36	44.5	25.21	0	5.33	7.72	6.69	7.94	4.89
Cashmere	15.46	22.67	7.4	46.48	2.72	40.96	42.8	28.33	7.09	0	7.36	8.36	5.11	2.38	6.58
Tile	26.84	26.48	7.32	61.94	32.87	61.8	51.97	40.94	29.3	31.87	4.04	0	0	6.73	1.54
Clay	35.93	26.88	31.36	62.2	17.16	58.78	48.42	8.67	30.57	31.04	10.21	12.21	24.36	19.96	7.46
Styrene foam	28.87	30.97	6.6	48.39	6.31	41.29	61.66	48.16	0	0	11.59	28.08	0	4.14	1.49
Nylon	25.88	32.96	6.14	34.81	2.27	32.12	76.42	56.96	5.94	0	1.88	3.46	0	2.69	0
Cork	30.72	33.79	8.67	29.29	1.56	33.19	45.45	40.82	6.09	0	1.42	1.37	0	4.47	0
Mesh (rough)	25.1	34.55	8.34	55.01	18.61	42.55	69.47	45.95	2.41	0	42.77	68.02	38.01	18.08	22.39
Broad cloth	29.34	34.85	9.29	33.81	2.4	39.41	67.07	60.94	4.07	0	3.26	3.52	0.29	0	2.11
Wood plate	32.06	35.12	7.88	22.15	0.24	13.49	41.25	33.42	14.32	9.77	0.78	0	0	4.97	0
Polishing sponge	36.95	36.01	7.96	40.65	3.03	29.75	78.1	51.1	12.91	1.52	14.3	28.27	4.09	26.95	2.33
Japanese paper	33.59	37.55	7.61	29.3	1.55	33.98	40.47	29.49	0.32	0	1.74	1.89	0	0	0
Cotton	17.25	39.18	6.12	45.05	11.69	39.53	64.73	56.01	3.76	0	4.9	4.9	4.3	0.19	4.02
Western paper	35.29	42.08	6.4	33.95	2.48	31.53	48.85	39.66	5.18	0	2.19	1.5	0	0	0.28
Fur	8.32	42.92	7.32	49.51	1.67	29.9	41.84	12.11	2.69	0	54.93	57.57	28.87	17.5	57.73
Mesh (fine)	31.89	42.98	7.9	42.06	5.67	39.38	64.72	50.85	3.12	0	43.17	75.13	41.13	17.72	15.28
Leather	36.71	44.49	9.67	29.79	1.62	34.83	38.34	27.17	15.23	2.19	3.99	3	2.04	4.98	0.09
Convex rubber	35.67	45.91	6.46	12.59	0.2	13.2	23.26	9.56	15.26	12.73	3.25	3.33	0	1.58	0
Artificial leather	23.82	58.09	8.3	27.89	0.66	25.56	39.59	25.97	10.23	0	5.07	8.55	7.2	9.7	2.42
Sponge rubber	47.61	60.92	8.17	31.96	0.76	27.43	64.19	53.76	2.86	0	14.39	43.41	14.59	18.26	6
Slime	63.47	68.07	150	67.86	33.45	47.11	54.76	20.16	23.53	11.42	54.1	40.86	79.06	66.27	90.68
Rubber	53.65	73.05	6.3	15.04	0.14	8.05	24.93	11.68	27.03	25.2	2.67	1.11	3.08	12.69	0
Artificial skin	34.87	92.7	28.57	45.75	4.03	31.65	62.17	42.01	22.82	21.05	20.28	48.69	36.94	41.25	25.96
Stainless plate	64.25	103.77	8.58	0.32	0.04	0	9.83	3.74	39.02	40.09	0	0.65	1.37	7.73	0
Low rebound sponge	13.24	105.18	11.35	15.5	0.21	10.39	45.26	44.44	2.01	4.99	92.31	78.6	44.84	15.8	27.28
Acrylic plate	55.15	111.22	8.95	7.42	0.13	9.5	11.41	10.97	22.12	17.4	0	0	1.3	0	0
Sticky tape	21.81	128.31	82.27	27.91	0.88	27.13	59.11	46.9	12.98	3.83	11.13	24.15	15.38	8.62	17.33
