# Supplementary material for: Predicting Sensory and Affective Tactile Perception from Physical Parameters Obtained by Using a Biomimetic Multimodal Tactile Sensor
Source: Sensors (Basel). 2024 Dec 30;25(1):147. doi: 10.3390/s25010147 (PMC11723034; doi:10.3390/s25010147)
Supplement: Supplementary file 1 [file sensors-25-00147-s001.zip › Table S2.pdf]

Table S2. Sensory descriptor scores for each material.

Words/materials	Bulky	Bumpy	Cold	Dry	Elastic	Firm	Hard	Moist	Rough	Scratch	Sleek	Slimy	Slippery	Smooth	Soft	Soggy	Sticky	Warm	Wet
Cream	1	1	4	1	3	4	1	5	1	1.5	4	5	4	5	4	5	5	2	5
Serum (after application)	1	1	4.5	1	2	3	2	4.5	1.5	1	4	5.5	4	5	3.5	4	4.5	2	5
Serum	2	2	4	2	3	4	2	5	2	1	4.5	5.5	5	5	5	5	4	2	5
Cream (after application)	1	1	2	1	3	4	1	5	1	1	5	6	4	5.5	4	5	5	2	5
Lotion (after application)	1	1	5	1	2	3	1.5	4.5	1.5	1.5	4	2	4	4	4	2	2	1	5
Lotion	1	1	5	2	2	2	2	5	1	2	4	2	4	4	3	2	2	2	5.5
Cashmere	2.5	3	2	4	2	2	2	2	3	3	4	1	2.5	4	4	2	2	4	2
Tile	4	5	5	5	1.5	1.5	5	2	4	3	4	1	3	2	2	1	1	2	2
Clay	2	2	4	2	5	5	4	5	2	3	3	4	4	4	4	5	5	2	5
Styrene foam	5	4	1	5	3	2	4	1	5	4	2	1	2	2	3	1	1	2	1
Nylon	3	2	2	3	2	1	3	2	2	2	4	1.5	4	3.5	2	1	1.5	2.5	2
Cork	4	4	2	5	2	2	5	1.5	4	3	2	1	3	3	2	1	1	3	2
Mesh (rough)	5	5	1	5	4	2	5	1	5	5	1	1	1	1	2	1	1	2	2
Broad cloth	5	3	2	4.5	2	1	4	2	4	2	2	1	2	3	3	1	1	2	1
Wood plate	2	1.5	2	4	1.5	1.5	5	2	2	2	4	1	4	4	2	1	1	4	2
Polishing sponge	6	5	2	5	2	2	5	2	6	6	1	1	1	1	2	1	1	3	2
Japanese paper	2	2	1.5	4	2	2	2	2	2	2	5	1.5	5	4	2	1	1	2	1.5
Cotton	4	1	1.5	4	1.5	2	2	2	3	3	4	1	2	3	4	1	1	2	1
Western paper	5	4	1	4	2	2	2	2	4	3	4	1	4	4	3	1	1	3	2
Fur	2	1.5	1	2	2	2	2	3	2	2	3	1	2	4	5	1	1	5	2
Mesh (fine)	2	2	2	3	5	4	2	4	2	2	3	1	3	4	5	2	1.5	4	1.5
Leather	2	4.5	2.5	4	2	3	3	4	3	3	3	1	3	3	3	2	2	3	2
Convex rubber	2	6	3	4	2	2	6	2	4	5	2	1	2	2	1	2	2	2	2
Artificial leather	2	2	2	2	2.5	3	2.5	4	2.5	1	4.5	1.5	4	5	4	2	2	3	2.5
Sponge rubber	2	2	2	3	5	4	4	5	2	3	4	2	3	4	4	2	2	3	4
Slime	1	2	5	1	4	4	2	4	1	2	3	5	4	4	5	5	5	2	5
Rubber	1.5	1	4	2	4	2	5	4	1	2	4.5	1.5	4	4	2	2.5	3	2	3
Artificial skin	1	1	3	1	5	5.5	2.5	5	1.5	2	4	1.5	4	4	4.5	4	3	2	4
Stainless plate	2	1	5	1	2	2	5	2	1	2	5	2	5	5	1	2	2	2	2
Low rebound sponge	2	1	2	2	5	5	1	5	2	2	4	1.5	4	4.5	6	3	2	2	3
Acrylic plate	1	1	4	1	1.5	1	5	2	1	1	5	1	6	4.5	1.5	1	2	2	1
Sticky tape	1	1	2	1	4	4	2	3	1	4	1	1	1	1.5	2	6	6	2	3
