# Supplementary material for: Predicting Sensory and Affective Tactile Perception from Physical Parameters Obtained by Using a Biomimetic Multimodal Tactile Sensor
Source: Sensors (Basel). 2024 Dec 30;25(1):147. doi: 10.3390/s25010147 (PMC11723034; doi:10.3390/s25010147)
Supplement: Supplementary file 1 [file sensors-25-00147-s001.zip › Table S3.pdf]

Table S3. Affective descriptor scores for each material.

Words/materials	Calm	Comfort	Delicate	Dislike	Fine	Friendly	Interest	Like	Luxury	Slight warmth	Pleasant	Unpleasant
Cream	2	4	3	2	3.5	4	2.5	3.5	3.5	2	4	2
Serum (after application)	3	3.5	2	2	3	3	2	2	2.5	2	3	3
Serum	3	4	4	2	2.5	2	2	2	3	3	4	3
Cream (after application)	2	3	3	2	3	4	3	3	2	2	3	4
Lotion (after application)	2	3	3	2.5	3	4	2	2	2	2	2.5	2.5
Lotion	2	3	3	2.5	2	2	2.5	2	2	2	3	2
Cashmere	4	5	3	2	4	4	2	3	3	3	4.5	1
Tile	2	3	3	2	3	2	2	2	2	2	3	2
Clay	3	4	3	3	3	3	3	3	3	2	4	3
Styrene foam	2	2	2	2	2	2	1	2	1	3	2	1
Nylon	2.5	3.5	3.5	2	3	3	3	4	3	3	4	2
Cork	2	3	2	2	3	2	2	2	3	3	3	1
Mesh (rough)	1.5	2	2.5	2	2	2	2	2	2	1	2	2
Broad cloth	2	3	2	2	3	2.5	1	3	2	3	2	2
Wood plate	4.5	4	2	2	3	4	3	4	3	4	4	2
Polishing sponge	3	2	2	3	3	2	2	2	2	2	2	2
Japanese paper	3	4	4	2	3	3	2	4	3	2	4	1
Cotton	2	3	4	2	2	2	2	3	2	2	3	1.5
Western paper	3	4	3	1	3	4	3	3	2	2	3	1
Fur	5	5	3	2	4	5	4	5	4	5	5	1
Mesh (fine)	4	4	3	2	3	4	3	4	3	4	4	2
Leather	3	5	3	1	4	4	2	3	3	3	3	2
Convex rubber	2	2	1	3	2	2	3	2	2	2	2	2
Artificial leather	3.5	4	4	2	4	4	2.5	4	4	3	4	2
Sponge rubber	3	4	3	2	4	3	3	3	3	2	4	2
Slime	4	4	2	2.5	3	3	4.5	4	3	2	5	4
Rubber	3	3	2	2	4	2	3	4	3	2	2	2
Artificial skin	3	4	3	2	4	4	3	4	4	3	5	2
Stainless plate	2	2	2	2	3	2	3	3	4	2	3	2
Low rebound sponge	4	4.5	4	1	4	4	3	4	4	3	5	1
Acrylic plate	3	3.5	3	2	4	2	3	3.5	3	2	4	2
Sticky tape	1	2	1.5	4	2	1.5	3	2	2	1.5	2	4.5
